# Torque Teno Virus Levels During Viral Respiratory Infections: The Interplay With Immune Dysregulation and Coagulopathy Biomarkers

**DOI:** 10.1002/jmv.70831

**Published:** 2026-02-07

**Authors:** Roberto Ferrarese, Pietro Giorgio Spezia, Sara Boutahar, Angelo Paolo Genoni, Gabriele Arcari, Gaia Zambon, Maria Dolci, Sara D'alessandro, Giuseppe Sberna, Serena Delbue, Nicasio Mancini, Fabrizio Maggi, Lucia Signorini, Federica Novazzi

**Affiliations:** ^1^ Department of Medicine and Technological Innovation University of Insubria Varese Italy; ^2^ Laboratory of Virology National Institute for Infectious Diseases, Lazzaro Spallanzani‐ IRCCS Rome Italy; ^3^ Department of Biomedical, Surgical and Dental Sciences University of Milano Milan Italy; ^4^ Ospedale di Circolo e Fondazione Macchi Laboratory of Medical Microbiology and Virology Varese Italy; ^5^ Department of Pharmacological and Biomolecular Sciences University of Milano Milan Italy

**Keywords:** cytokine, endothelial dysfunction, IL‐6, immunity biomarker, influenza, interferon response, respiratory viral infections, rhinovirus, RSV, thrombosis, Torquetenovirus, TTV

## Abstract

Torque teno virus (TTV) is a ubiquitous, nonenveloped DNA virus of the Anelloviridae family and a proposed surrogate marker of immune competence. Although nonpathogenic, its replication reflects host immune status and is associated with immune dysregulation during respiratory viral infections (RVIs). This study evaluated the interplay among TTV levels, inflammatory, endothelial, and coagulation biomarkers in acute RVIs. We collected 468 leftover material samples (234 respiratory and 234 blood samples) from hospitalized patients with PCR‐confirmed RVIs. Patients were stratified by viral etiology, differential involvement of the respiratory tract, age, and possible co‐detected pathogens. Cytokines (IL‐6, IL‐8, IL‐1β, TNF‐α), IFNs (α/β/γ), and endothelial markers (ICAM‐1, VCAM‐1) were quantified using microfluidic immunoassays. Routine coagulation parameters were measured in a subset of patients. TTV DNA load was quantified in both compartments using real‐time PCR. Associations with inflammatory and coagulation parameters were assessed using nonparametric tests. TTV DNA was detectable across all age groups and viral etiologies, with higher levels in infants (0–1 years) and elderly patients (81–94 years). Blood and respiratory TTV levels were strongly correlated (*r* = 0.53, *p* < 0.0001). In infants, blood TTV correlated positively with IL‐6 and CRP; in elderly patients, inverse correlations with TNF‐α, IFN‐α, and ICAM‐1 suggested less regulated antiviral and endothelial responses. No significant differences were found by viral type or possible co‐detected pathogens, though cytokine–TTV associations persisted. TTV levels reflect systemic and local immune activation during RVIs and deserve further investigation as possible noninvasive biomarker of immune dysregulation and thromboinflammatory risk. Longitudinal studies are needed to determine its prognostic value.

## 1 Background

*Torque teno virus* (TTV) is a small, nonenveloped, circular single‐stranded DNA virus belonging to the *Anelloviridae* family and is considered part of the normal human virome [1, 2, 3, 4]. Although TTV is generally regarded as nonpathogenic, its replication dynamics are closely linked to the host immune status. Elevated TTV DNA levels are frequently observed in immunocompromised individuals, such as transplant recipients and patients with HIV, suggesting that TTV replication reflects the degree of immune suppression [5, 6, 7].

Recent studies have detected TTV DNA in respiratory specimens from patients with acute viral infections, including influenza viruses (Flu), respiratory syncytial virus (RSV), and severe acute respiratory syndrome coronavirus 2 (SARS‐CoV‐2), where high TTV loads have been associated with a dysregulated immune response and increased disease severity [8, 9, 10, 11, 12]. Analogously, higher TTV loads have been linked to severe disease outcomes and hyperinflammatory states characterized by increased IL‐6 and C‐reactive protein (CRP) levels [13, 14]. As a matter of fact, TTV appears to interact with the host immune system through modulation of cytokine expression, influencing both pro‐ and anti‐inflammatory pathways [15]. Increased TTV replication has been correlated with elevated levels of proinflammatory cytokines such as interleukin (IL)‐6, IL‐1β, tumor necrosis factor‐α (TNF‐α), and IL‐8, as well as the anti‐inflammatory cytokine IL‐10 [5, 10, 16, 17, 18, 19].

Collectively, these findings support the potential utility of TTV load as a noninvasive biomarker for monitoring immune competence and predicting clinical outcomes in patients with respiratory viral infections (RVIs).

The main objective of this project is to implement a multidisciplinary approach to investigate the role of TTV levels as biomarker in severe RVIs caused by FLU, RSV, human rhinovirus (HRV), and parainfluenza virus/adenovirus (PIV/ADV), with a focus on mechanisms that trigger systemic inflammation and thrombotic events.

We collected leftover material from respiratory and plasma samples of hospitalized patients with RVIs and tested for a broad panel of inflammatory, endothelial, and coagulation biomarkers. In parallel, TTV levels were quantified both in respiratory and blood samples.

## 2 Materials and Methods

### 2.1 Study Population

A total of 468 samples (234 respiratory and 234 plasma samples) were collected from patients admitted to the Respiratory Physiopathology Unit, the intensive care unit, the neonatal intensive care unit, and the Pediatric Ward of the University Hospital of Varese (Italy) between September 2024 and March 2025. Leftover materials were used to perform the study, thus not requiring any institutional review board approval. All patients presented with respiratory symptoms and tested positive to a RV on *n* = 204 nasopharyngeal swabs and/or on *n* = 30 bronchoalveolar lavage (BAL). Due to the lack of detailed clinical and radiological data on all patients, all cases requesting diagnostic testing on BAL were considered as lower respiratory tract infections, which were used as a criterion to distinguish between severe and nonsevere infections. The characteristics of subjects are summarized in Table 1.

**Table 1:** Study population.

	Subgroup		*n* (%)
Gender	M		138 (59)
	F		96 (41)
Age range	0–1		36 (15.4)
	2–4		30 (12.8)
	5–17		31 (13.2)
	19–59		38 (16.2)
	60–80		57 (24.4)
	81–94		42 (18)
Infecting virus	FLU 116 (49.6%)	UTR	97 (83.6)
		LTR	19 (16.4)
	RSV 52 (22.2%)	UTR	46 (88.5)
		LTR	6 (11.5)
	HRV 51 (21.8%)	UTR	46 (90.2)
		LTR	5 (9.8)
	ADV/PIV 15 (6.4%)	UTR	15 (100)
Codetection	Viral	Present	82 (35)
		Absent	152 (65)
	Bacterial	Present	75 (32)
		Absent	100 (43)
		N/A	59 (25)
			Mean ± SE (UoM)
TTV levels	Blood		4.65 ± 2.44 log/mL
	Respiratory samples		3.92 ± 1.93 log/ng DNA

### 2.2 Molecular Testing of Respiratory Samples and Blood Laboratory Parameters

The molecular testing for RV was performed afternucleic acid extraction from respiratory samples using the SeegeneAllplex^TM^ Respiratory panel (Arrow diagnostics), a comprehensive assay for the detection and identification of 26 pathogens using one‐step real‐time polymerase chain reaction (RT‐PCR). Mor in details, the assay is, composed of four different panels, including Panel 1 analytes are: Flu A, Flu B, RSV A, RSV B ‐ Flu A‐H1, Flu A‐H1pdm09, Flu A‐H3; Panel 2 analytes are: hdV, human enterovirus, Parainfluenza virus 1 (PIV 1), Parainfluenza virus 2 (PIV 2), Parainfluenza virus 3 (PIV 3), Parainfluenza virus 4 (PIV 4), Metapneumovirus (MPV); Panel 3 analytes are: Bocavirus (HBoV), Rhinovirus (HRV), Coronavirus NL63 (CoV NL63), Coronavirus 229E (CoV 229E), Coronavirus OC43 (CoV OC43) and SARS‐CoV‐2; Panel 4 analytes are: *Bordetella parapertussis, Bordetella pertussis, Chlamydophila pneumoniae, Haemophilus influenzae, Legionella pneumophila, Mycoplasma pneumoniae*, and *Streptococcus pneumoniae*.

Peripheral venous blood samples were also collected at the same time as the respiratory specimen was taken upon admission to the ER for baseline biochemical analyses. All biochemical parameters, including CRP, prothrombin time (PT), activated partial thromboplastin time (APTT) test and fibrinogen were measured in a subpopulation of our cohort as part of routine laboratory analysis using standard laboratory methods.

Whole blood was collected into commercially available tubes containing ethylene diamine tetraacetic acid (EDTA) on the day of confirmed PCR diagnosis of RV infection. Blood samples were centrifuge at 1000–2000 × g for 10 min. The resulting plasma was collected, divided into 0.5 mL aliquots, and stored at –20°C or lower until analysis. Samples that were haemolyzed, icteric, or lipemic were excluded from the study. Stored plasma samples include *n* = 234 from RV‐positive patients and *n* = 22 from a healthy control group.

### 2.3 Automated Immunoassay for Chemokine and Cytokine Plasma Assessment

Plasma levels of IL‐6, IL‐8, IFN‐α, TNF‐α, IFN‐γ, IL‐1β, IFN‐β, ICAM‐1, VCAM‐1 were measured in triplicate using ELLA™ microfluidic immunoassays (Protein Simple, Bio‐Techne, USA) according with the manufacturer's specifications. Detection limits were: IL‐6 (0.626 pg/mL), IL‐8 (0.4 pg/mL), IFN‐α (3.79 pg/mL), TNF‐α (6.23 pg/mL), IFN‐γ (2.56 pg/mL), IL‐1β (1 pg/mL), IFN‐β (2.88 pg/mL), and ICAM‐1 and VCAM‐1 (1.26 pg/mL). Intra and interassay coefficients of variation were all below 11%. Data were analyzed using the Bio‐Plex Manager software version 6.0 (Bio‐Rad Laboratories, USA).

### 2.4 TTV DNA Detection and Quantification in Blood and Respiratory Samples

Viral DNA was extracted from whole blood samples using QIAamp DNA Blood mini kit (Qiagen GmbH, Germany) according to the manufacturer's instructions. TTV DNA presence and load were assessed using a single‐step, in‐house TaqMan PCR assay, as described elsewhere [8]. The assay, referred to as “universal PCR,” employs forward and reverse primers targeting a highly conserved segment of the untranslated region of the viral genome's UTR and quantifies the total TTV DNA load without distinguishing among different TTV species present in a single subject's blood. The lower detection limit was 1.0 Log copies of TTV DNA per mL of blood. Methods for copy number quantification, as well as evaluations of specificity, sensitivity, intra and interassay precision, and reproducibility, have been previously outlined [20]. The method used to analyze TTV in respiratory samples is the same as that used for blood samples, but the results are reported as log/ng extracted DNA [8].

### 2.5 Statistical Analysis

Results are reported as mean ± standard error. The analysis of the measured TTV levels in the patient cohort, which was stratified first by infecting virus and then by age groups, involved two separate approaches. Quade's non‐parametric ANCOVA was used to address the uneven age distribution in infecting virus sorted groups, while the Kruskal–Wallis test, followed by Dunn's multiple comparison test, was used for age range sorted groups. Correlations were determined using Spearman's correlation. For the analyses that required it, we have reported Hedges' g (Hg) to measure the effect size. Results were considered statistically significant for *p*‐values < 0.05. Statistical analyses were performed with Prism V8.0 (GraphPad Software Inc., La Jolla, CA) and IBM SPSS Statistics Version 31 (IBM, Armonk, NY, USA).

## 3 Results

### 3.1 Correlation Between TTV Levels in Blood and Respiratory Samples in the General Cohort

**Figure 1:**
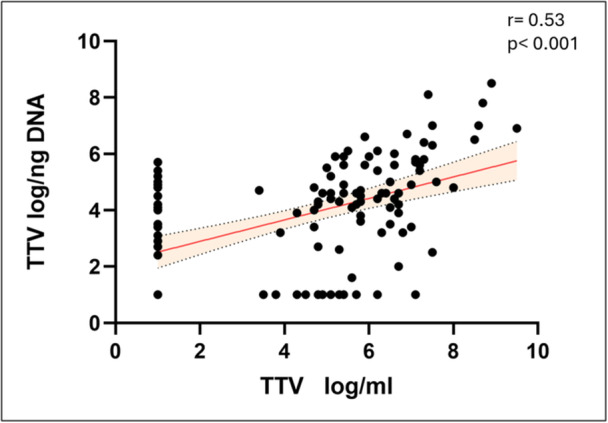
Correlation between serum and respiratory samples TTV levels in general population. Each dot represents a patient. Linear regression (continuous lines) and 95% confidence interval (dashed line and shaded area) are depicted. Spearman correlation coefficients (*r*) and *p*‐value (*p*) are indicated.

First, we evaluated the correlation between blood and respiratory samples TTV levels, and with cytokines and blood parameters were then assessed. In the general population, a statistically significant direct correlation between TTV levels in blood and TTV levels in the respiratory tract (*r* = 0.53, *p* < 0.001) was observed (Figure 1). A complete summary of the correlations observed in the general population is presented in Table 2A.

**Table 2:** Correlation matrix calculated between pairs of variables in the general population (A) and in populations stratified by age range (B). Spearman correlation coefficients (*r*) and *p*‐value (*p*) are reported.

A				
General population				
Spearman *r*	TTV log/mL	*p*‐value	TTV log/ng DNA	*p*‐value
Age	0.035	0.5797	−0.112	0.2348
CRP	0.028	0.6559	−0.090	0.3495
TTV log/mL			0.530	0.0001
TTV log/ng DNA	0.530	0.0001		
IFN‐γ (pg/mL)	−0.015	0.8110	0.090	0.3434
IFN‐α (pg/mL)	−0.056	0.3684	−0.019	0.8415
TNF‐α (pg/mL)	0.058	0.3583	0.046	0.6274
IL‐6 (pg/mL)	0.087	0.1671	0.111	0.2411
IL‐8 (pg/mL)	0.005	0.9346	−0.075	0.4258
IFN‐β (pg/mL)	−0.008	0.8947	−0.059	0.5342
IL‐1β (pg/mL)	0.045	0.4766	−0.054	0.5703
ICAM‐1 (pg/mL)	−0.062	0.3202	−0.078	0.4121
VCAM‐1 (pg/mL)	−0.014	0.8284	−0.038	0.6917
PT	0.118	0.2034	−0.062	0.6434
APTT	0.041	0.6554	−0.116	0.3821
P‐INR	0.107	0.2464	−0.053	0.6910
FIBRINOGEN	0.035	0.8654	−0.108	0.6978

**B**														
**0–1**					**2–4**					**5‐17**				
Spearman *r*	TTV log/mL	*p*‐value	TTV log/ng DNA	*p*‐ value	Spearman *r*	TTV log/mL	*p*‐value	TTV log/ng DNA	*p* value	Spearman r	TTV log/mL	*p* value	TTV log/ng DNA	*p* value
CRP	0.377	0.0280	0.371	0.1179	CRP	0.118	0.5353	−0.099	0.7385	CRP	−0.138	0.4826	−0.613	0.0288
TTV log/mL			0.705	0.0005	TTV log/mL			0.878	0.0001	TTV log/ml			−0.024	0.9315
TTV ns	0.705	0.0005			TTV ns	0.878	0.0001			TTV ns	−0.024	0.9315		
IFN‐γ (pg/mL)	0.174	0.3107	0.055	0.8168	IFN‐γ (pg/mL)	0.008	0.9663	−0.046	0.8796	IFN‐γ (pg/mL)	−0.221	0.2313	−0.049	0.8574
IFN‐α (pg/mL)	0.203	0.2358	0.107	0.6539	IFN‐α (pg/mL)	0.199	0.2926	0.266	0.3573	IFN‐α (pg/mL)	0.075	0.6893	−0.346	0.1874
TNF‐α (pg/mL)	0.221	0.1953	0.084	0.7255	TNF‐α (pg/mL)	0.129	0.4964	0.037	0.9002	TNF‐α (pg/mL)	0.198	0.2864	−0.354	0.1769
IL‐6 (pg/mL)	0.364	0.0290	0.384	0.0950	IL‐6 (pg/mL)	−0.015	0.9373	−0.147	0.6158	IL‐6 (pg/mL)	0.044	0.8136	−0.284	0.2832
IL‐8 (pg/mL)	0.182	0.2874	−0.009	0.9696	IL‐8 (pg/mL)	−0.023	0.9046	−0.297	0.3025	IL‐8 (pg/mL)	0.142	0.4465	−0.052	0.8487
IFN‐β (pg/mL)	0.104	0.5478	−0.052	0.8279	IFN‐β (pg/mL)	−0.340	0.0658	−0.471	0.0907	IFN‐β (pg/mL)	0.298	0.1038	0.066	0.8052
IL‐1β (pg/mL)	−0.021	0.9038	−0.281	0.2304	IL‐1β (pg/mL)	0.220	0.2438	0.111	0.7034	IL‐1β (pg/mL)	−0.150	0.4193	−0.278	0.2935
ICAM‐1 (pg/mL)	0.243	0.1533	0.246	0.2952	ICAM‐1 (pg/mL)	−0.340	0.0656	−0.262	0.3656	ICAM‐1 (pg/mL)	0.136	0.4655	−0.560	0.0262
VCAM‐1 (pg/mL)	0.195	0.2552	0.063	0.7922	VCAM‐1 (pg/mL)	−0.305	0.1007	−0.411	0.1458	VCAM‐1 (pg/mL)	0.136	0.4648	−0.252	0.3436
PT					PT					PT	0.119	0.7618	−0.400	0.7500
APTT					APTT					APTT	−0.060	0.8852	−0.632	0.5000
P‐INR					P‐INR					P‐INR	0.119	0.7618	−0.400	0.7500
FIBRINOGEN					FIBRINOGEN					FIBRINOGEN	0.029	1.0000	−0.500	1.0000

**19–59**					**60–80**					**81‐94**				
Spearman *r*	TTV log/mL	*p*‐value	TTV log/ng DNA	*p*‐value	Spearman r	TTV log/mL	*p*‐value	TTV log/ng DNA	p value	Spearman r	TTV log/mL	p value	TTV log/ng DNA	p value
CRP	−0.053	0.7525	−0.586	0.0489	CRP	−0.069	0.5518	−0.034	0.8593	CRP	0.359	0.0195	0.474	0.0260
TTV log/mL			0.447	0.1443	ttv log/ml			0.449	0.0129	ttv log/ml			0.312	0.1581
TTV ns	0.447	0.1443			ttv ns	0.449	0.0129			ttv ns	0.312	0.1581		
IFN‐γ (pg/mL)	0.252	0.1264	0.196	0.5364	IFN‐γ (pg/mL)	−0.069	0.5464	−0.002	0.9929	IFN‐γ (pg/mL)	−0.207	0.1894	−0.037	0.8689
IFN‐α (pg/mL)	−0.078	0.6426	0.243	0.4417	IFN‐α (pg/mL)	−0.106	0.3506	−0.053	0.7817	IFN‐α (pg/mL)	−0.487	0.0011	−0.250	0.2627
TNF‐α (pg/mL)	−0.050	0.7645	0.450	0.1423	TNF‐α (pg/mL)	0.067	0.5554	0.034	0.8584	TNF‐α (pg/mL)	−0.328	0.0337	0.034	0.8808
IL‐6 (pg/mL)	0.121	0.4676	0.232	0.4622	IL‐6 (pg/mL)	0.014	0.9026	0.080	0.6726	IL‐6 (pg/mL)	0.107	0.4996	0.096	0.6702
IL‐8 (pg/mL)	−0.158	0.3432	0.276	0.3804	IL‐8 (pg/mL)	−0.043	0.7070	−0.202	0.2837	IL‐8 (pg/mL)	−0.065	0.6829	0.171	0.4470
IFN‐β (pg/mL)	0.027	0.8725	0.093	0.7678	IFN‐β (pg/mL)	0.074	0.5190	−0.139	0.4644	IFN‐β (pg/mL)	−0.125	0.4285	−0.091	0.6866
IL‐1β (pg/mL)	0.122	0.4641	−0.193	0.5424	IL‐1β (pg/mL)	−0.053	0.6443	0.062	0.7438	IL‐1β (pg/mL)	0.042	0.7925	0.164	0.4665
ICAM‐1 (pg/mL)	−0.333	0.0412	0.094	0.7695	ICAM‐1 (pg/mL)	−0.014	0.9015	−0.187	0.3217	ICAM‐1 (pg/mL)	−0.307	0.0484	−0.251	0.2593
VCAM‐1 (pg/mL)	−0.319	0.0510	0.516	0.0886	VCAM‐1 (pg/mL)	0.077	0.5029	0.019	0.9208	VCAM‐1 (pg/mL)	−0.172	0.2769	−0.011	0.9601
PT	−0.290	0.1203	−0.342	0.3618	PT	−0.157	0.3095	−0.103	0.6225	PT	0.318	0.0811	−0.111	0.6679
APTT	0.149	0.4399	−0.454	0.2171	APTT	−0.110	0.4700	−0.151	0.4721	APTT	0.208	0.2533	0.144	0.5784
P‐INR	−0.268	0.1528	−0.342	0.3618	P‐INR	−0.188	0.2151	−0.099	0.6390	P‐INR	0.324	0.0752	−0.101	0.6975
FIBRINOGEN	−0.064	0.8933			FIBRINOGEN	0.226	0.5570	0.580	0.2444	FIBRINOGEN				

*Note:* The red, yellow and green colors represent a scale indicating the relative magnitude of the Spearman's coefficient for each feature.

### 3.2 Analysis of TTV Levels in Blood and Respiratory Samples in the Patient Cohort Stratified by Infecting Virus and Correlation With Cytokine Levels and Blood Parameters

**Figure 2:**
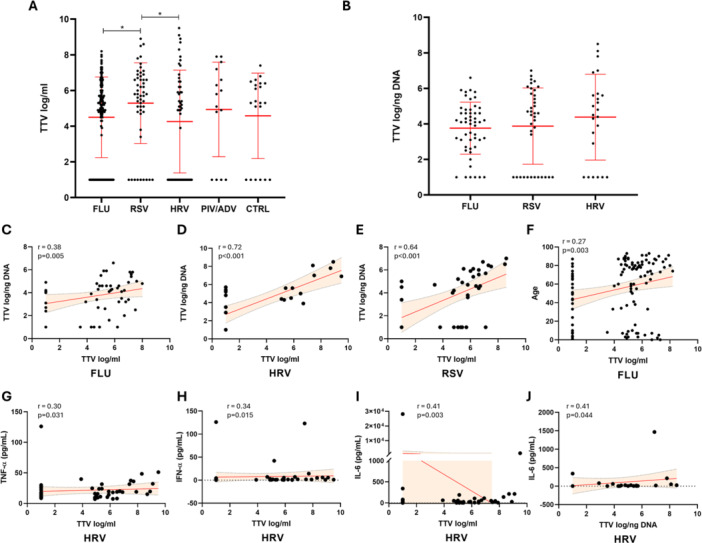
(A, B) Serum (A) and respiratory samples (B) TTV levels measured in our cohort of patients sorted by infecting virus. Median with range is depicted. Statistical analysis: Quade's nonparametric ANCOVA (*p < 0.05). (C–J) Correlations of serum with respiratory TTV levels in patients infected by FLU (C), HRV (D), and RSV (E). Correlation of serum TTV levels and age in patients infected by FLU (F). Correlations of blood TTV levels with TNF‐a (G), IFN‐a (H), and IL‐6 (I). Correlation of respiratory TTV with IL‐6 (J). Each dot represents a patient. Linear regression (continuous lines) and 95% confidence interval (dashed line and shaded area) are depicted. Spearman correlation coefficients (*r*) and *p*‐value (*p*) are indicated.

We stratified our patient cohort by infecting virus and evaluated the differences in blood TTV levels among the groups of patients infected by different respiratory viruses, and between infected patients and the control group. Statistically significant differences were observed between FLU and RSV (FLU: 4.5 ± 0.21 log/mL; RSV: 5.3 ± 0.31 log/mL; *p* = 0.01) and between RSV and HRV (HRV: 4.3 ± 0.4 log/mL; *p* = 0.03). No statistically significant differences were observed involving blood TTV levels of Adeno/Para (4.9 ± 0.68 log/mL) and control group (CTRL) (4.6 ± 0.52 log/mL) samples. The analysis of TTV levels in respiratory samples was possible only among patients infected with FLU, RSV, and HRV. In this comparison, no statistically significant differences were found among the groups (FLU: 3.8 ± 0.2 log/ng; RSV: 3.9 ± 0.35 log/ng; HRV 4.4 ± 0.49 log/ng) (Figure 2A,B). Performing the correlation analysis, we observed a statistically significant direct correlation between blood TTV and TTV in respiratory samples infected with FLU (*r* = 0.38; *p* = 0.005), RSV (*r* = 0.64; *p* < 0.001) and HRV (*r* = 0.72; *p* < 0.001) (Figure 2C–E). Furthermore, in FLU‐infected samples a direct correlation between blood TTV and age (*r* = 0.27; *p* = 0.003) was observed. On the contrary, in HRV positive samples, a direct correlations between blood TTV levels and TNF‐α (*r* = 0.3; *p* = 0.031) and IFN‐α (*r* = 0.34; *p* = 0.015) levels was observed. Furthermore, both blood and respiratory samples TTV levels directly correlated with IL‐6 levels (*r* = 0.41; *p* = 0.003 and *r* = 0.41; *p* = 0.04 respectively) (Figure 2F–J). No statistically significant correlations regarding blood and respiratory samples TTV levels were observed in the RSV, Adeno/Para, and control cohorts. All correlations observed in the population stratified by infecting virus are reported in (Table S1).

### 3.3 Analysis of TTV Levels in Blood and Respiratory Samples in the Patient Cohort Stratified by Age and Correlation With Cytokine Levels and Blood Parameters

**Figure 3:**
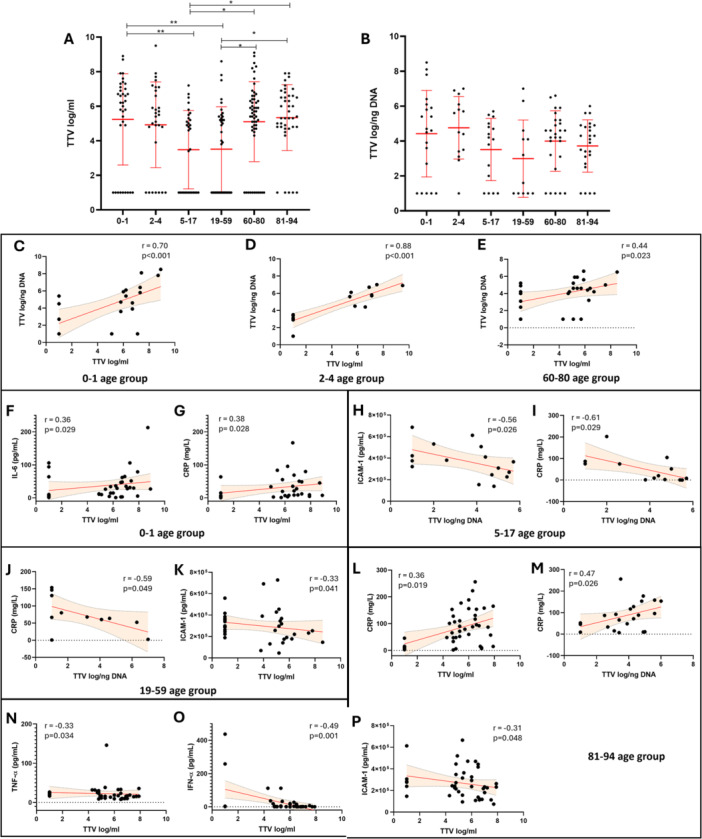
(A, B) Serum (A) and respiratory samples (B) TTV levels measured in our cohort of patients sorted by age range. Median with range is depicted. Statistical analysis: Kruskal–Wallis test followed by Dunn's multiple comparison test (*p < 0.05; **p < 0.01). (C–E) Correlation between serum and respiratory samples TTV levels in 0–1 (C), 2–4 (D), and 60–80 (E) age groups. (F–G) Correlations of serum TTV levels with IL‐6 (F) and CRP (G) in the 0–1 age range population. (H‐I) Correlations of respiratory samples TTV levels with ICAM‐1 (H) and CRP (I) in the 5–17 age group population. (J, K) Correlations of respiratory samples TTV levels with CRP (J) and of serum TTV levels with ICAM‐1 (K) in 19–59 age group. (L–P) Correlations of respiratory samples TTV levels with CRP (L) and of serum TTV levels with CRP (M), TNF‐a (N), IFN‐a (O), and ICAM‐1 (P) in the 81–94 age group population. Each dot represents a patient. Linear regression (continuous lines) and 95% confidence interval (dashed line and shaded area) are depicted. Spearman correlation coefficients (*r*) and *p*‐value (*p*) are indicated.

Subsequently, we performed the same analysis in the patient cohort stratified by age groups. Higher blood TTV levels in the 0–1 age group compared to the 5–17 and 19–59 groups (0–1: 5.2 ± 0.44 log/mL; 5–17: 3.5 ± 0.41 log/mL; 19–59: 3.5 ± 0.4 log/mL; *p* = 0.006 and *p* = 0.005 respectively) were observed. Furthermore, blood TTV levels were significantly higher in the oldest age group (81–94) years: (5.3 ± 0.3 log/mL) compared to both the pediatric (5–17 years) and the younger adult (19–59 years) groups (both *p* = 0.028). Finally, the 60–80 years age group (5.1 ± 0.31 log/mL) also showed significantly higher TTV levels when compared to the 5–17 and 19–59 age groups (both *p* = 0.038). Unlike the blood samples, TTV levels in respiratory samples stratified by age groups showed no statistically significant differences (0–1: 4.4 ± 0.49 log/ng; 2–4: 4.4 ± 0.49 log/ng; 5–17: 4.4 ± 0.49 log/ng; 19–59: 4.4 ± 0.49 log/ng; 60–80: 4.4 ± 0.49 log/ng; 81–94: 4.4 ± 0.49 log/ng) (Figure 3A,B). The correlation analysis evidenced a direct correlation between blood and respiratory samples TTV levels in the age groups 0–1 (*r* = 0.7; *p*< 0.001), 2–4 (*r* = 0.88; *p* < 0.001) and 60–80 (*r* = 0.44; *p* = 0.023) (Figure 3C–E). In the 0–1 age range a direct correlations of blood TTV levels with IL‐6 (*r* = 0.36; *p* = 0.029) and with CRP (*r* = 0.38; *p* = 0.028) (Figure 3F,G) was observed. In 5–17 age range, an inverse correlation of respiratory samples TTV levels with ICAM‐1 (*r* = −0.56; *p* = 0.026) and with CRP (*r* = −0.61; *p* = 0.029) Figure 3H,I) was observed. Same inverse correlation between respiratory samples TTV levels and CRP was also found in the 19–59 age group (*r* = −0.58; *p* = 0.048) and additionally, an inverse correlation between blood TTV and ICAM‐1 (*r* = −0.33; *p* = 0.41) was also observed (Figure 3J,K). Finally, in 81–94 age group a direct correlations of CRP with both blood (*r* = 0.36; *p* = 0.019) and respiratory samples (*r* = 0.47; *p* = 0.026) TTV levels was observed, while an inverse correlations where observed between blood TTV levels, TNF‐α (*r* = −0.33; *p* = 0.034), IFN‐α (*r* = −0.49; *p* = 0.001) and ICAM‐1 levels (*r* = −0.31; *p* = 0.048) (Figure 3L–P). All direct and inverse correlations observed in the population stratified by age groups are reported in Table 2B.

### 3.4 Analysis of TTV Levels in Blood and Respiratory Samples in the Patient Cohort Stratified by Infection Site and Correlation With Cytokine Levels and Blood Parameters

**Figure 4:**
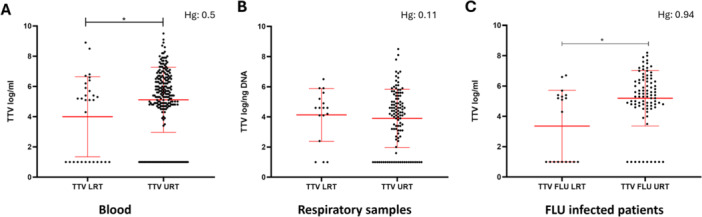
(A, B) Serum (A) and respiratory samples (B) TTV levels measured in the overall population, irrespective of the infecting virus, and stratified by LRT or URT involvement. (C) TTV levels measured in the FLU infected population, stratified by LRT or URT involvement. Each dot represents a patient. Median with range and Hedges' g (Hg) are represented. Statistical analysis: Mann–Whitney test (*p < 0.05).

TTV levels in the URT and LRT samples were analyzed, showing a statistically significant difference in blood TTV levels (LRT: 4 ± 0.48 log/mL; URT: 5.1 ± 0.14 log/mL; *p* = 0.04) while no significant difference was observed in respiratory samples (LRT: 4.1 ± 0.42 log/ng; URT: 3.9 ± 0.18 log/ng) (Figure 4A,B). Higher blood TTV levels were observed in the samples from the URT (LRT: 3.4 ± 0.54 log/mL; URT: 5.2 ± 0.2 log/mL; CTRL: 4.6 ± 0.52 og/mL; LRT vs. URT *p* = 0.02) when considering the FLU virus positive group although the small sample size limits the power of this analysis (Figure 4C). No statistically significant differences were observed in blood TTV levels infected with RSV (LRT: 6.1 ± 1.2 log/mL; URT: 5.3 ± 0.32 log/mL) and HRV (LRT: 3.8 ± 1.2 log/mL; URT: 5 ± 0.25 log/mL) (data not shown). We then analyzed cytokine levels in the LRT and URT subgroups and observed that IL‐6 and IL‐8 were significantly higher in the LRT compared to the URT subgroup (IL‐6: LRT (304 ± 861 pg/mL) vs. URT (197 ± 1978 pg/mL), *p* < 0.001; IL‐8: LRT (135 ± 118 pg/mL) vs. URT (100 ± 181 pg/mL), *p* < 0.001) (Figure S1A,B). We then performed a correlation analysis comparing the TTV levels in both blood and respiratory samples, correlating them with each other and with cytokine levels, within the LRT and URT subgroups. In both subgroups, we observed a statistically significant direct correlation between blood and respiratory samples TTV levels (LRT: *r* = 0.53, *p* = 0.03; URT: *r* = 0.57, *p* < 0.001) (Figure S1C,D). No statistically significant correlations were detected between TTV levels in blood or respiratory samples and the measured cytokines.

### 3.5 TTV Levels in Bacterial and/or Viral Codetections

**Figure 5:**
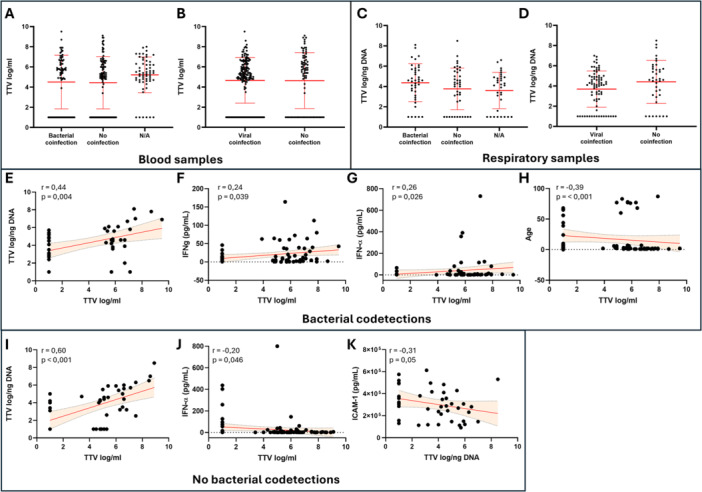
(A, D) Serum TTV levels compared between the patient population with bacterial (A) or viral (B) codetections and patients with no codetections. Respiratory samples TTV levels compared between the patient population with bacterial (C) or viral (D) codetections and patients with no codetections. Each dot represents a patient. Statistical analysis: Mann‐Whitney test. (E–K) Correlations of serum TTV levels with respiratory TTV (E), IFN‐a (F), IFN‐g (G) and age (H) observed in the population with bacterial codetections. Correlations of serum TTV with respiratory TTV (I) and IFN‐a (J) and correlation of respiratory TTV with ICAM‐1 (H) in the patient cohort without bacterial codetections. Each dot represents a patient. Linear regression (continuous lines) and 95% confidence interval (dashed line and shaded area) are depicted. Spearman correlation coefficients (*r*) and *p*‐value (*p*) are indicated.

**Figure 6:**
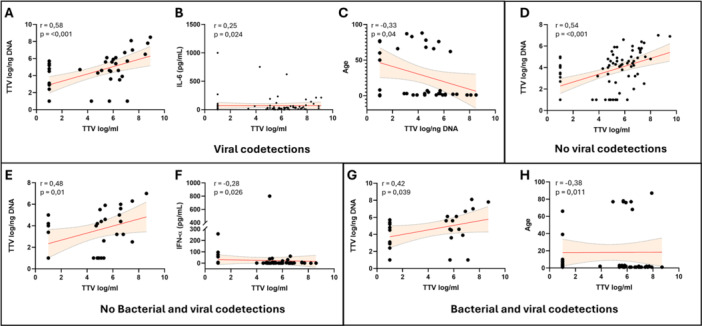
(A–D) Correlations of serum TTV levels with respiratory TTV (A) and IL‐6 (B), and correlation of respiratory TTV with age (C) observed in the population with viral codetections. Correlation between serum and respiratory TTV in the patient cohort without viral codetections (D). (E–H) Correlations of blood TTV with respiratory TTV (E) and IFN‐a (F) in patient cohort without any type of codetections and with respiratory TTV (G) and age (H) in patient cohort with both viral and bacterial codetections. Each dot represents a patient. Linear regression (continuous lines) and 95% confidence interval (dashed line and shaded area) are depicted. Spearman correlation coefficients (*r*) and *p*‐value (*p*) are indicated.

Finally, samples were grouped according to the presence of bacterial and/or viral codetections. No statistically significant differences in serum and respiratory TTV levels were observed when comparing samples with bacterial codetections to samples without them (blood TTV: 4.5 ± 0.31 log/mL vs. 4.4 ± 0.26 log/mL; respiratory samples TTV: 4.4 ± 0.3 log/ng vs. 3.8 ± 0.32 log/ng) and samples with viral codetections compared to those without them (blood TTV: 4.7 ± 0.18 log/mL vs. 4.6 ± 0.31 log/mL; respiratory samples TTV: 3.7 ± 0.21 log/ng vs. 4.4 ± 0.35 log/ng) (Figure 5A–D). In patients with bacterial codetections, statistically significant direct correlations between blood and respiratory TTV levels (*r* = 0.44, *p* = 0.004), IFN‐α (*r* = 0.26; *p* = 0.02) and IFN‐γ plasma levels (*r* = 0.24; *p* = 0.04) were observed, while an inverse correlation was observed with patient's age (*r* = −0.39; *p* < 0.001) (Figure 5E–H). In the cohort without bacterial codetections, a direct correlation between blood and respiratory TTV levels (*r* = 0.6; *p* < 0.001) was observed, while inverse correlations were found between blood TTV and IFN‐α levels (*r* = −0.2; *p* = 0.046) and between respiratory TTV and ICAM‐1 levels (*r* = −0.31; *p* = 0.05) (Figure 5I‐K). In the general population with viral codetections, a direct correlations between blood and respiratory TTV levels (*r* = 0.58; *p* < 0.001) and between blood TTV and IL‐6 plasma levels (*r* = 0.25; *p* = 0.024) were observed, while an inverse correlation was present between respiratory TTV levels and age (*r* = −0.33; *p* = 0.04). A direct correlation between blood and respiratory TTV levels was also present in the general population without viral codetections (*r* = 0.54; *p* < 0.001) (Figure 6A–D). Finally, samples were grouped according to the presence or absence of both types of codetections, viral and bacterial. No statistically significant differences were observed between the different groups for both TTV levels in the blood and those measured in respiratory samples. In the subgroup of samples without viral and bacterial codetections, a direct correlation between blood and respiratory TTV levels (*r* = 0.48; *p* = 0.01) and an inverse correlation between blood TTV and plasma IFN‐α levels (*r* = −0.28; *p* = 0.026) were observed (Figure 6E,F). Finally, in the subgroup with both viral and bacterial correlations, a direct correlation between blood and respiratory TTV levels (*r* = 0.42; *p* = 0.039) and an inverse correlation between blood TTV levels and age (*r* = −0.38; *p* = 0.011) was observed (Figure 6G,H).

## 4 Discussion

This study provides a comprehensive evaluation of TTV levels in both respiratory and blood compartments of patients with acute RVIs, exploring their correlation with inflammatory and coagulation markers. Our results reinforce the concept that TTV, while not directly pathogenic, reflects host immune status and may serve as a dynamic biomarker of immune dysregulation during viral infections.

Consistent with previous observations, we found that TTV DNA levels were detectable across all age groups and infection types, but they varied significantly with age and with the specific infecting RSV [5, 8, 10, 15]. Higher TTV levels were observed in infants (0–1 year) and elderly patients (81–94 years), both of whom represent populations with relatively immature or senescent immune systems, respectively. This age‐dependent bimodal distribution underscores the influence of immune competence on TTV replication dynamics and supports the notion that TTV levels mirror host immune homeostasis rather than specific viral tropism [6, 7].

Importantly, a significant correlation was observed between TTV levels in blood and respiratory samples, suggesting that systemic and local replication are interconnected. This correlation was particularly strong in RSV and HRV‐infected patients, aligning with evidence that severe or prolonged respiratory infections may enhance systemic immune perturbations, allowing greater TTV replication [9, 11]. These data support the view that TTV can act as a surrogate marker of immune activation in both local and systemic compartments during acute infection.

When analyzed in relation to inflammatory mediators, TTV levels showed distinct age‐ and virus‐specific patterns of association. In the youngest cohort, positive correlations between blood TTV, IL‐6, and CRP indicate that increased TTV replication parallels systemic inflammation and acute‐phase responses. This finding mirrors prior studies in COVID‐19 and influenza, where higher TTV loads were linked to hyperinflammatory states and elevated cytokine production [10, 13, 21]. In older adults, the observed inverse correlations between blood TTV and TNF‐α, IFN‐α, and ICAM‐1 suggest that immune senescence and reduced antiviral cytokine responses may facilitate viral persistence and higher TTV replication. Collectively, these results support a bidirectional interaction: impaired immune control promotes TTV replication, while persistent TTV may further modulate cytokine balance, perpetuating immune dysregulation.

The observed associations between TTV and endothelial activation markers (ICAM‐1, VCAM‐1) provide additional insight into the link between viral infection, inflammation, and coagulopathy. Endothelial dysfunction is a central feature of severe respiratory infections, including influenza and SARS‐CoV‐2, and is driven by inflammatory cytokines, complement activation, and viral invasion of endothelial tissue [22, 23, 24]. Our findings of inverse correlations between TTV and ICAM‐1 in several age groups may reflect compensatory endothelial responses or differential timing of inflammatory and viral load peaks. These interactions warrant further study to determine whether TTV load could indicate endothelial integrity or the propensity toward microvascular dysfunction in acute infection.

The analysis of co‐detections did not reveal significant differences in absolute TTV levels, suggesting that the mere presence of additional pathogens does not necessarily amplify TTV replication. However, in subgroups with bacterial or viral co‐detections, correlations between TTV and cytokines such as IFN‐α, IFN‐γ, and IL‐6 imply that TTV replication remains sensitive to the overall inflammatory milieu. The inverse relationship observed between TTV and age in co‐detections patients further emphasizes that immune competence modulates TTV control more strongly than pathogen diversity itself.

Taken together, our data support a conceptual model in which TTV levels represent a functional “marker” of host immune balance. Elevated TTV in blood reflects diminished immune surveillance or sustained immune activation, both of which can coexist in severe respiratory viral disease. By mirroring the host's antiviral and inflammatory status, TTV may provide an integrative biomarker bridging the gap between immune dysregulation and the downstream vascular or coagulative complications that drive severe disease.

Future studies should explore whether longitudinal monitoring of TTV load can predict disease progression, treatment response, or risk of thromboinflammatory complications in patients with RVIs. Integration of TTV quantification into clinical algorithms, particularly when combined with cytokine or endothelial biomarkers, may enhance risk stratification and early therapeutic decision‐making.

This study demonstrates that TTV levels in both blood and respiratory samples correlate with immune and inflammatory dynamics during RVIs. While absolute TTV concentrations did not differ significantly between viral etiologies, their modulation by age, cytokine pattern, and systemic inflammatory status highlights TTV as a sensitive indicator of immune competence.

The observed relationships between TTV, IL‐6, interferons, and endothelial activation markers suggest that TTV may participate in/or at least mirror the complex interplay between immune dysregulation and endothelial dysfunction that underlies severe RVI pathogenesis. Our findings reinforce the potential of TTV load as a noninvasive biomarker to monitor immune activation, predict clinical severity, and guide management of inflammation‐associated coagulopathy in RVIs. The study limitation include the fact that respiratory virus Ct values weren't available.

Further research using longitudinal sampling, mechanistic models, and multi‐omics approaches is warranted to define whether TTV acts solely as a bystander of immune perturbation or contributes directly to the modulation of host inflammatory and thrombotic pathways.

## Supporting information


**Supporting Figure 1:** (A, B) IL‐6 (A) and IL‐8 (B) levels measured in patient cohort stratified by infection site (LRT vs. URT). Each dot represents a patient. Median with range and Hedges’ g (Hg) are represented. Statistical analysis: Mann‐Whitney test (***p < 0.001). (C, D) Correlation between serum and respiratory samples TTV levels in LRT (C) and URT (D) subgroups. Each dot represents a patient. Linear regression (continuous lines) and 95% confidence interval (dashed line and shaded area) are depicted. Spearman correlation coefficients (*r*) and *p* value (*p*) are indicated.

**Supporting Table 1:** Correlation matrix calculated between pairs of variables in samples stratified by infecting virus. Spearman correlation coefficients (r) and p values are reported.

## Data Availability

The data that support the findings of this study are available from the corresponding author upon reasonable request. The raw data supporting the conclusions of this article will be made available by the authors upon request.
