# Supplementary figures and images for: Torque Teno Virus Levels During Viral Respiratory Infections: The Interplay With Immune Dysregulation and Coagulopathy Biomarkers
Source: J Med Virol. 2026 Feb 7;98(2):e70831. doi: 10.1002/jmv.70831 (PMC12882057; doi:10.1002/jmv.70831)

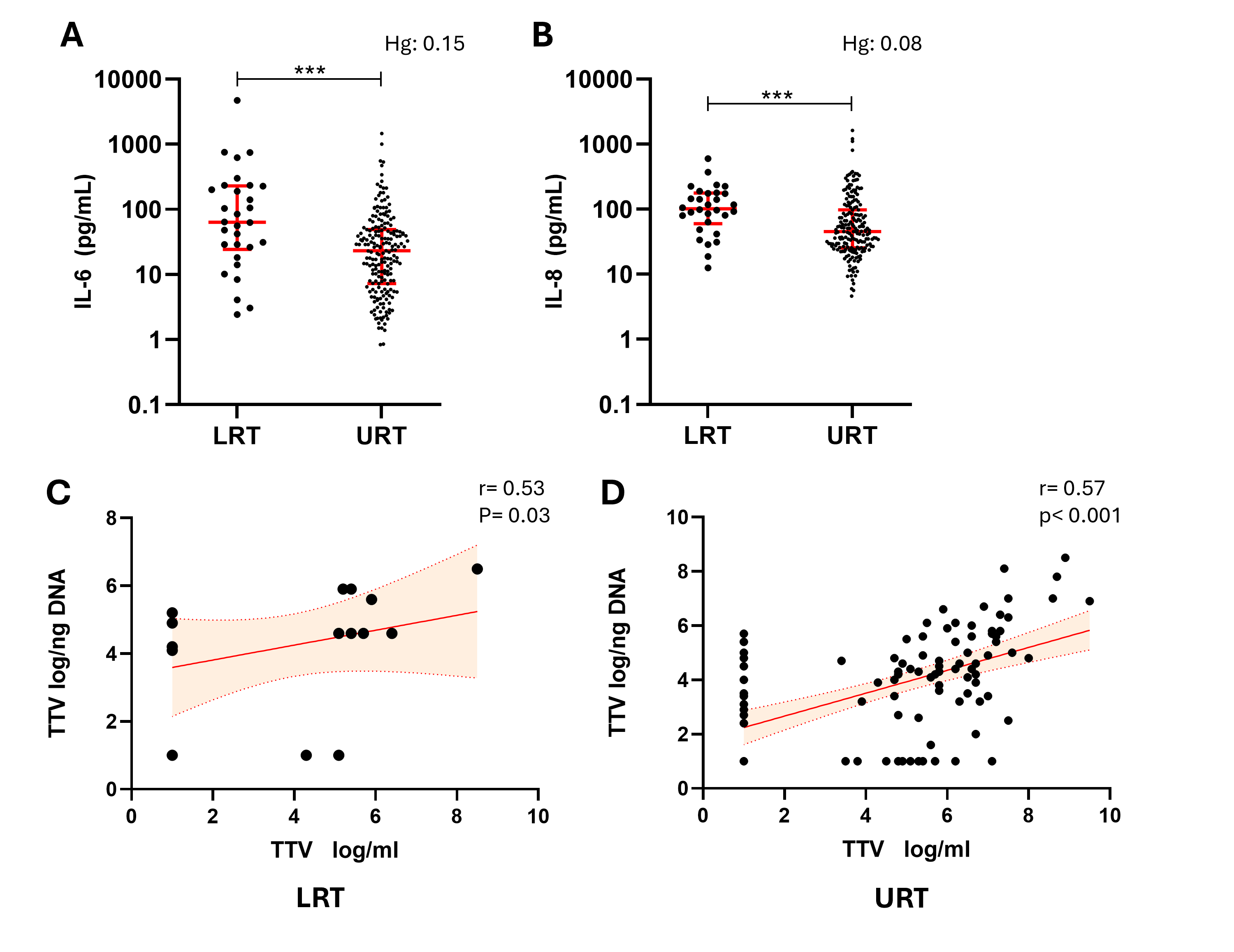

Supplement: Supplementary file 1 — Supporting Figure 1: (A, B) IL‐6 (A) and IL‐8 (B) levels measured in patient cohort stratified by infection site (LRT vs. URT). Each dot represents a patient. Median with range and Hedges’ g (Hg) are represented. Statistical analysis: Mann‐Whitney test (***p < 0.001). (C, D) Correlation between serum and respiratory samples TTV levels in LRT (C) and URT (D) subgroups. Each dot represents a patient. Linear regression (continuous lines) and 95% confidence interval (dashed line and shaded area) are depicted. Spearman correlation coefficients (r) and p value (p) are indicated. [file JMV-98-e70831-s001.tif]
