# Supplementary material for: Torque Teno Virus Levels During Viral Respiratory Infections: The Interplay With Immune Dysregulation and Coagulopathy Biomarkers
Source: J Med Virol. 2026 Feb 7;98(2):e70831. doi: 10.1002/jmv.70831 (PMC12882057; doi:10.1002/jmv.70831)
Supplement: Supplementary file 2 — Supporting Table 1: Correlation matrix calculated between pairs of variables in samples stratified by infecting virus. Spearman correlation coefficients (r) and p values are reported. [file JMV-98-e70831-s002.docx]

**Supplementary Table 1** Correlation matrix calculated between pairs of variables in samples stratified by infecting virus. Spearman correlation coefficients (r) and p values are reported.

| **FLU** | | | | |  | **HRV** | | | | |
| --- | --- | --- | --- | --- | --- | --- | --- | --- | --- | --- |
| Spearman r | TTV log/mL | p value | TTV log/ng DNA | p value |  | Spearman r | TTV log/mL | p value | TTV log/ng DNA | p value |
| AGE | 0.273 | 0.0030 | 0.084 | 0.5521 |  | AGE | 0.033 | 0.8169 | -0.240 | 0.8169 |
| CRP | 0.051 | 0.5929 | 0.040 | 0.7839 |  | CRP | 0.009 | 0.9485 | 0.042 | 0.9485 |
| TTV log/mL |  |  | 0.384 | 0.0050 |  | TTV log/mL |  |  | 0.720 | 0.0001 |
| TTV log/ng DNA | 0.384 | 0.0050 |  |  |  | TTV log/ng DNA | 0.720 | 0.0001 |  |  |
| IFN-γ (pg/mL) | -0.118 | 0.2054 | 0.050 | 0.7231 |  | IFN-γ (pg/mL) | 0.120 | 0.4004 | 0.348 | 0.0961 |
| IFN-α (pg/mL) | -0.169 | 0.0701 | -0.105 | 0.4577 |  | IFN-α (pg/mL) | 0.340 | 0.0148 | 0.291 | 0.1677 |
| TNF-α (pg/mL) | -0.084 | 0.3713 | 0.150 | 0.2879 |  | TNF-α (pg/mL) | 0.302 | 0.0311 | 0.214 | 0.3149 |
| IL-6 (pg/mL) | -0.043 | 0.6492 | 0.048 | 0.7357 |  | IL-6 (pg/mL) | 0.411 | 0.0027 | 0.415 | 0.0438 |
| IL-8 (pg/mL) | 0.037 | 0.6899 | 0.078 | 0.5825 |  | IL-8 (pg/mL) | 0.088 | 0.5383 | -0.080 | 0.7095 |
| IFN-β (pg/mL) | -0.005 | 0.9545 | -0.138 | 0.3278 |  | IFN-β (pg/mL) | 0.020 | 0.8913 | 0.083 | 0.7000 |
| IL-1β (pg/mL) | 0.110 | 0.2419 | 0.097 | 0.4937 |  | IL-1β (pg/mL) | -0.002 | 0.9899 | -0.229 | 0.2822 |
| ICAM-1 (pg/mL) | -0.179 | 0.0539 | -0.100 | 0.4799 |  | ICAM-1 (pg/mL) | 0.037 | 0.7967 | 0.106 | 0.6204 |
| VCAM-1 (pg/mL) | -0.066 | 0.4831 | 0.116 | 0.4129 |  | VCAM-1 (pg/mL) | 0.127 | 0.3751 | 0.096 | 0.6570 |
| PT | 0.123 | 0.2911 | 0.178 | 0.3138 |  | PT | 0.192 | 0.4568 | -0.649 | 0.1230 |
| APTT | 0.163 | 0.1558 | 0.182 | 0.3041 |  | APTT | -0.250 | 0.3290 | -0.270 | 0.5587 |
| P-INR | 0.099 | 0.3918 | 0.179 | 0.3105 |  | P-INR | 0.186 | 0.4719 | -0.649 | 0.1230 |
| FIBRINOGEN | 0.051 | 0.8642 | 0.429 | 0.3536 |  | FIBRINOGEN | -0.505 | 0.2548 | -0.600 | 0.4167 |
|  |  |  |  |  |  |  |  |  |  |  |
| **RSV** | | | | |  | **ADV/PIV** | | | | |
| Spearman r | TTV log/mL | p value | TTV log/ng DNA | p value |  | Spearman r | TTV log/mL | p value | TTV log/ng DNA | p value |
| AGE | -0.212 | 0.1321 | -0.055 | 0.7435 |  | AGE | -0.045 | 0.8732 |  |  |
| CRP | 0.041 | 0.7761 | -0.122 | 0.4640 |  | CRP | 0.321 | 0.2823 |  |  |
| TTV log/mL |  |  | 0.641 | 0.0000 |  | TTV log/mL |  |  |  |  |
| TTV log/ng DNA | 0.641 | 0.0000 |  |  |  | TTV log/ng DNA |  |  |  |  |
| IFN-γ (pg/mL) | -0.036 | 0.8026 | -0.001 | 0.9944 |  | IFN-γ (pg/mL) | -0.052 | 0.8533 |  |  |
| IFN-α (pg/mL) | -0.121 | 0.3911 | 0.064 | 0.7010 |  | IFN-α (pg/mL) | -0.271 | 0.3262 |  |  |
| TNF-α (pg/mL) | 0.033 | 0.8177 | -0.039 | 0.8165 |  | TNF-α (pg/mL) | 0.503 | 0.0581 |  |  |
| IL-6 (pg/mL) | 0.103 | 0.4670 | 0.086 | 0.6097 |  | IL-6 (pg/mL) | 0.188 | 0.4996 |  |  |
| IL-8 (pg/mL) | -0.083 | 0.5595 | -0.142 | 0.3944 |  | IL-8 (pg/mL) | 0.238 | 0.3892 |  |  |
| IFN-β (pg/mL) | -0.080 | 0.5741 | -0.047 | 0.7773 |  | IFN-β (pg/mL) | 0.251 | 0.3604 |  |  |
| IL-1β (pg/mL) | 0.054 | 0.7025 | -0.187 | 0.2608 |  | IL-1β (pg/mL) | 0.204 | 0.4610 |  |  |
| ICAM-1 (pg/mL) | 0.038 | 0.7911 | -0.229 | 0.1674 |  | ICAM-1 (pg/mL) | -0.162 | 0.5599 |  |  |
| VCAM-1 (pg/mL) | 0.188 | 0.1874 | -0.091 | 0.5929 |  | VCAM-1 (pg/mL) | -0.099 | 0.7234 |  |  |
| PT | -0.097 | 0.6849 | -0.324 | 0.1903 |  | PT | 0.400 | 0.5167 |  |  |
| APTT | -0.208 | 0.3785 | -0.459 | 0.0551 |  | APTT | 0.100 | 0.9500 |  |  |
| P-INR | -0.082 | 0.7327 | -0.297 | 0.2318 |  | P-INR | 0.400 | 0.5167 |  |  |
| FIBRINOGEN | -0.200 | 0.9167 | -1.000 | 0.0833 |  | FIBRINOGEN |  |  |  |  |
